# Novel machine learning approach toward classification model of HIV-1 integrase inhibitors†

**DOI:** 10.1039/d4ra02231a

**Published:** 2024-05-02

**Authors:** Tieu-Long Phan, The-Chuong Trinh, Van-Thinh To, Thanh-An Pham, Phuoc-Chung Van Nguyen, Tuyet-Minh Phan, Tuyen Ngoc Truong

**Affiliations:** a Bioinformatics Group, Department of Computer Science, and Interdisciplinary Center for Bioinformatics, Universität Leipzig Härtelstraße 16-18 04107 Leipzig Germany; b Department of Mathematics and Computer Science, University of Southern Denmark Odense M DK-5230 Denmark; c Faculty of Pharmacy, Grenoble Alpes University La Tronche 38700 France; d Falcuty of Pharmacy, University of Medicine and Pharmacy at Ho Chi Minh City Ho Chi Minh City 700000 Vietnam truongtuyen@ump.edu.vn

## Abstract

HIV-1 (human immunodeficiency virus-1) has been causing severe pandemics by attacking the immune system of its host. Left untreated, it can lead to AIDS (acquired immunodeficiency syndrome), where death is inevitable due to opportunistic diseases. Therefore, discovering new antiviral drugs against HIV-1 is crucial. This study aimed to explore a novel machine learning approach to classify compounds that inhibit HIV-1 integrase and screen the dataset of repurposing compounds. The present study had two main stages: selecting the best type of fingerprint or molecular descriptor using the Wilcoxon signed-rank test and building a computational model based on machine learning. In the first stage, we calculated 16 different types of fingerprint or molecular descriptors from the dataset and used each of them as input features for 10 machine-learning models, which were evaluated through cross-validation. Then, a meta-analysis was performed with the Wilcoxon signed-rank test to select the optimal fingerprint or molecular descriptor types. In the second stage, we constructed a model based on the optimal fingerprint or molecular descriptor type. This data followed the machine learning procedure, including data preprocessing, outlier handling, normalization, feature selection, model selection, external validation, and model optimization. In the end, an XGBoost model and RDK7 fingerprint were identified as the most suitable. The model achieved promising results, with an average precision of 0.928 ± 0.027 and an F1-score of 0.848 ± 0.041 in cross-validation. The model achieved an average precision of 0.921 and an F1-score of 0.889 in external validation. Molecular docking was performed and validated by redocking for docking power and retrospective control for screening power, with the AUC metrics being 0.876 and the threshold being identified at −9.71 kcal mol^−1^. Finally, 44 compounds from DrugBank repurposing data were selected from the QSAR model, then three candidates were identified as potential compounds from molecular docking, and PSI-697 was detected as the most promising molecule, with *in vitro* experiment being not performed (docking score: −17.14 kcal mol^−1^, HIV integrase inhibitory probability: 69.81%)

## Abstract 2

HIV-1 (human immunodeficiency virus-1) has been causing severe pandemics by attacking the immune system of its host.

## Graphical abstract

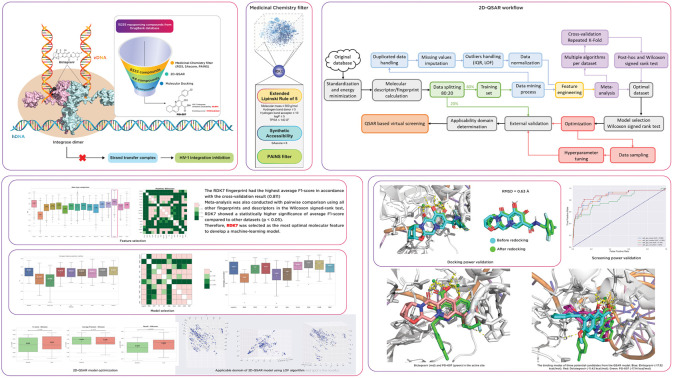

## 1. Introduction

According to the UNAIDS 2021 statistics (United Nations Joint Programme on HIV/AIDS),^1^ there were more than 38.4 million people worldwide living with HIV. Since HIV was first discovered in the 1980s, the disease has caused about 34.7 million deaths. However, there are no specific drugs or vaccines, so individuals living with HIV can only be treated with antiviral therapy like antiretroviral drugs (ARV), suppressing symptoms and slowing down the process leading to AIDS. Following several HIV-1 treatment regimens, clinical therapy should incorporate multiple ARV drugs to ensure the antiviral effect and reduce the risk of drug resistance. Therefore, many ARV drugs have been studied and developed, including reverse transcriptase inhibitors comprising both nucleoside^2^ and non-nucleoside inhibitors,^3^ protease inhibitors,^4^ integrase inhibitors^5^ and fusion inhibitors.^6^ Regarding protein targets, the integrase (IN) enzyme stands as a prominent target for medicinal chemistry researchers.^7^ This enzyme is produced by a virus with reverse transcription, a process in which viral nucleic acids are catalyzed to form covalent bonds between its genetic information and the DNA (deoxyribonucleic acid) of the host's infected cells.^8^ Thus, inhibition of integrase during strand transfer can prevent viral proliferation and, therefore, prolong the host's lifetime. These inhibitory compounds are called integrase strand transfer inhibitors (INSTIs), and often combine IN inhibitors with other HIV medicines to mitigate drug resistance.^9^

Machine learning has revolutionized many fields, including drug discovery. In this field, AI (artificial intelligence) has been used to create predictive models for ADMET, drug response,^10^ toxicity,^11^ and anticancer activity.^12^ These models allow virtual screening and prediction of compound activity.^13^ Several studies have been conducted on building models based on machine learning to predict the activity of compounds against IN, including those by A. Kurczyk *et al.* (2015), Y. Li *et al.* (2017), and L. A. Machado *et al.* (2022).^14–16^ In contrast to traditional QSAR models,^17^ which solely focused on linear equations to correlate the molecular descriptor with biological activities, the machine learning approach enables the implementation of non-linear models for QSAR.

This study aimed to discover promising candidates for organic synthesis plans by building computational models and using those models to screen the dataset of repurposing compounds from the DrugBank database for potential IN inhibitors. In this study, a novel approach was taken to select fingerprints or descriptors using the Wilcoxon signed-rank test.^18^ Likewise, the model selection process for determining the optimal model was conducted differently from the approach used by Y. Li *et al.* (2017).^14^ The study of Y. Li *et al.* utilized ECFP_4 fingerprint as the model input along with Support Vector Machine, Decision Tree, Function Tree, and Random Forest for machine learning model.

## 2. Methods

**Fig. 1:**
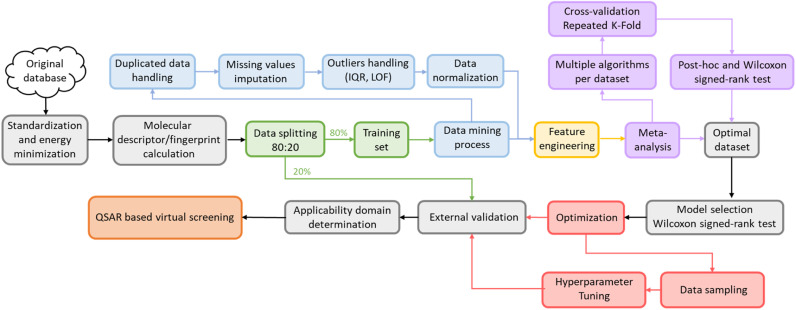
Model development pipeline includes two stages: dataset selection and model establishment.

This study was carried out using Python 3.8 with AMD Ryzen 93900X CPU core consisting of 12 processors, 3.79 GHz processor speed, 500 GB memory, and 96.0 GB RAM operating on Linux 22.04. Molecular descriptors and fingerprints were generated *via* open-source packages, including Padel 2.21 Descriptor,^14^ RDKit 2020.3.1,^19^ Mordred 1.2.0,^20^ Map 4 1.0,^21^ and MHFP.^22^ The machine learning model was completed using scikit-learn 1.1.1 library^23^ with the steps described in the diagram below (Fig. 1). All stages of the study were conducted with the same random state (value = 42) to ensure reproducibility. The source code, all datasets, and the results of this study are available at: https://github.com/Medicine-Artificial-Intelligence/HIV_IN_Classification.

### 2.1. Dataset

8979 molecules inhibiting HIV-1 integrase were collected from the ChEMBL 33 database. Biological activity standardization was performed, including the target organism being “human immunodeficiency virus 1”, assay type being “B type”, and “IC50” value in measurement unit columns, followed by canonicalizing SMILES structures. Upon completion of these stages, 2834 compounds remained in the dataset for building the model. Besides, 15 235 structures collected from the DrugBank database were prepared for virtual screening, with repurposing and repositioning strategies.

### 2.2. Optimal dataset selection

SMILES notations were converted into molecules to calculate 16 types of fingerprints or descriptors, called molecular features, including 3D-Mordred, RDKit descriptor, Mol2vec, MACCS, PubChem, Avalon, ECFP2, ECFP4, ECFP6, RDK5, RDK6, RDK7, Cats2D, 2D-Pharmacophore Gobbi (Ph4), MAP4, and SECFP (raw_data_features). The data preparation process was performed for all molecular features set, including target normalization with the threshold of pIC50 being 7 (meaning active or class 1 if pIC50 is equal or above 7, and inactive or class 0 for the counterpart), dataset division (80 : 20) with stratification principle resulted in 1995 compounds in the training set and 499 compounds in the external validation set with the imbalance ratio of 0.404 between the active and inactive classes.

Then, the data mining process was conducted on the training set and similar methods were applied the external validation set. First, 1995 compounds with molecular features underwent data removal to eliminate duplicate rows and columns, followed by missing values handling utilizing KNNImputer from the scikit-learn library (only for the 3D-Mordred dataset) before ending up with low variance removal using a threshold of 0.05. Next, the Local Outlier Factor (LOF) technique was employed with a parameter setting of “n_neighbors = 20” to remove outliers in the training set and novelty in the external validation set. The final step in the data mining process is data normalization using a rescaling method, in which MinMaxScaler was applied to map all data into the range [0,1].

Feature extraction was executed using algorithms from the Random Forest (RF) algorithm, followed by applying 10 different machine learning algorithms to the molecular features: Logistic Regression (Logistic), k-Nearest Neighbor (KNN), Support Vector Machine (SVM), Random Forest (RF), Extra Tree (ExT), Adaboost (Ada), Gradient Boosting (Grad), XGBoost (XGB), CatBoost (CatB), and Multilayer Perceptron (MLP). A meta-analysis was conducted to identify the optimal feature set, employing the Wilcoxon signed-rank test and utilizing a 3 × 10 Repeated Stratified K-Fold cross-validation approach with the F1-score serving as the primary evaluation metric. To account for multiple comparisons in the Wilcoxon test, the Holm^24^ method was applied, as indicated by the p_adjust = ‘holm’ parameter in the scikit-posthocs package,^25^ crucial for controlling the family-wise error rate amidst numerous pairwise comparisons. The Holm method systematically adjusts each *p*-value from the pairwise tests, reducing false positives and enhancing the validity of the significant results, illustrating a detailed statistical analysis approach and ensuring the reliability of the molecular feature set selection findings.

### 2.3. Machine learning model development

The most effective molecular features set selected above underwent similar data processing and mining steps but experienced a slight difference in the feature extraction stage. Instead of just using Random Forest to select essential features, eight different methods, consisting of Chi-squared (Chi2), Mutual information (Mutual_info), Random Forest (RF), Extra Tree (ExT), Adaboost (Ada), Gradient Boosting (Grad), XGBoost (XGB), and Logistic Regression (Logistic) were performed to compare the performance of these models with the baseline model, for which the feature extraction was not performed. The optimal algorithm was then used to reduce the data dimensionality.

The reduced-dimensional data was performed for model selection to select an optimal algorithm for model development. Ten different algorithms were selected for this step, including Logistic Regression (Logistic), k-Nearest Neighbor (KNN), Support Vector Machine (SVM), Random Forest (RF), Extra Tree (ExT), Adaboost (Ada), Gradient Boosting (Grad), XGBoost (XGB), CatBoost (CatB), and Multilayer Perceptron (MLP). 3 × 10 RepeatedStratifiedKFold cross-validation with Wilcoxon signed-rank test^26^ was also performed for these two stages.

Moreover, the Tree-structured Parzen Estimator (TPE) algorithm from the Optuna library was utilized for Bayesian Optimization (BO) to optimize the hyperparameters. BO is a global optimization technique that builds a surrogate model of the objective function and uses an acquisition function to suggest the next sample point.^27,28^

### 2.4. Model evaluation

The performance of a model can be evaluated by its learnability from data and generalizability on unseen datasets, performed through internal and external validation, respectively. Internal validation (IV) involves cross-validation techniques for training models and hyperparameter tuning. External validation (EV), on the other hand, utilizes a validation dataset from an independent source to assess the model's performance unbiasedly. As such, the results of EV provide crucial evidence for the generalizability of a QSAR model.^29^

The models' performance in this study was evaluated using statistical parameters such as F1-score, average precision, precision, and recall. Precision is calculated as the ratio of true positive predictions to the sum of true positive and false positive predictions.^30^
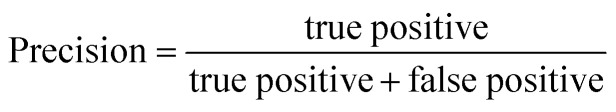


Recall is a statistical measure that quantifies the proportion of true positive instances that are correctly identified by a predictive model.^30^
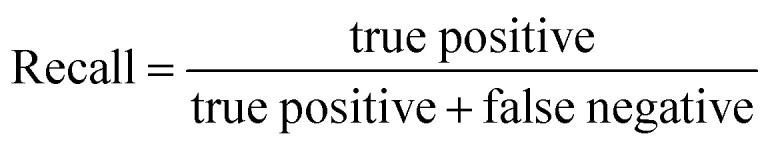


Average Precision (AP) is calculated as the weighted mean of precision at each threshold, the weight is the increase in recall from the prior threshold.^31^
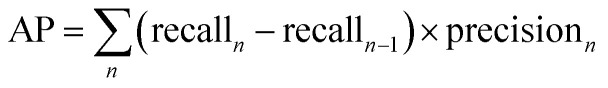


The F1-score is calculated as the harmonic mean of precision and recall, providing a measure of the trade-off between them.^30^
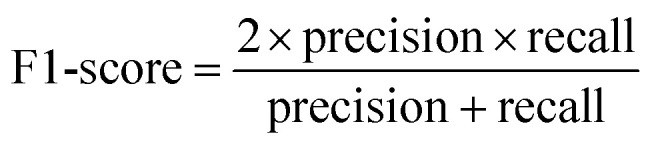


### 2.5. Molecular docking

In this study, we retrieved the structure of HIV Integrase from the Protein Data Bank (PDB ID: 6PUW),^32^ and split it into ligand and protein components. For protein preparation, we extracted the protein's .pdb file (PDB ID: 6PUW), removed the co-crystal ligand and water molecules, and retained only the protein chains. Hydrogens and Gasteiger charges were added to the protein using MGLTools 1.5.7, converting it into a .pdbqt file. In parallel, ligand preparation involved converting compounds in SMILES format into 2D structures using RDKit's *MolFromSmiles*, then transforming these into 3D conformations using the *EmbedMolecule* module with a random seed of 42. After energy minimization using the MMFF94 force field through *MMFFOptimizeMolecule*, capped at 10 000 iterations, the ligands were saved in .pdb format. Further preparation with MGLTools 1.5.7 added hydrogens and Gasteiger charges, resulting in the final.pdbqt files for the ligands. Finally, the grid box was defined as a cube of 60 × 60 × 60 grid points, with coordinates *x* = 143.399 Å, *y* = 159.402 Å, and *z* = 177.382 Å. Molecular docking was performed using AutoDock-GPU, employing specific parameters to guide the process. The seed parameter was set to a random number seed of 42 to ensure reproducibility. We used 1000 runs of the Lamarckian genetic algorithm (*nrun*) to thoroughly explore the potential binding configurations. Additionally, a maximum of 2 × 10^9^ score evaluations per LGA run (*nev*) was specified, allowing an extensive assessment of the docking interactions within each run.

The performance of the docking model was validated by redocking to validate docking power (RMSD ≤2 Å) and retrospective control (enrichment analysis) to validate screening power. DeepCoy was utilized to generate decoy (active: decoy = 1 : 50) for the latter step,^33^ and the performance was measured by the Receiver Operating Characteristic (ROC) curve and Area Under the Curve (AUC). Additionally, the Geometric Mean (G-Mean) was employed to determine the optimal cut-off point for the ROC curve.^34^ The G-Mean is a metric measuring the balance between classification performance for majority and minority classes.

## 3. Results

### 3.1. Molecular features set selection

**Fig. 2:**
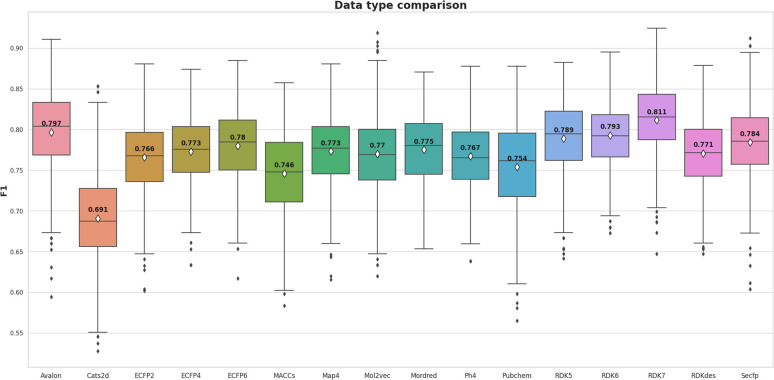
The meta-analysis of 16 types of fingerprints and descriptors utilizing F1-score metric.

The meta-analysis utilized the Wilcoxon signed-rank test to identify the optimal features set. The F1-score was the primary metric used to compare the performance of 16 molecular feature sets. The evaluation results are detailed in Table S1 (ESI),† while the summarized comparison is illustrated in Fig. 2.

**Fig. 3:**
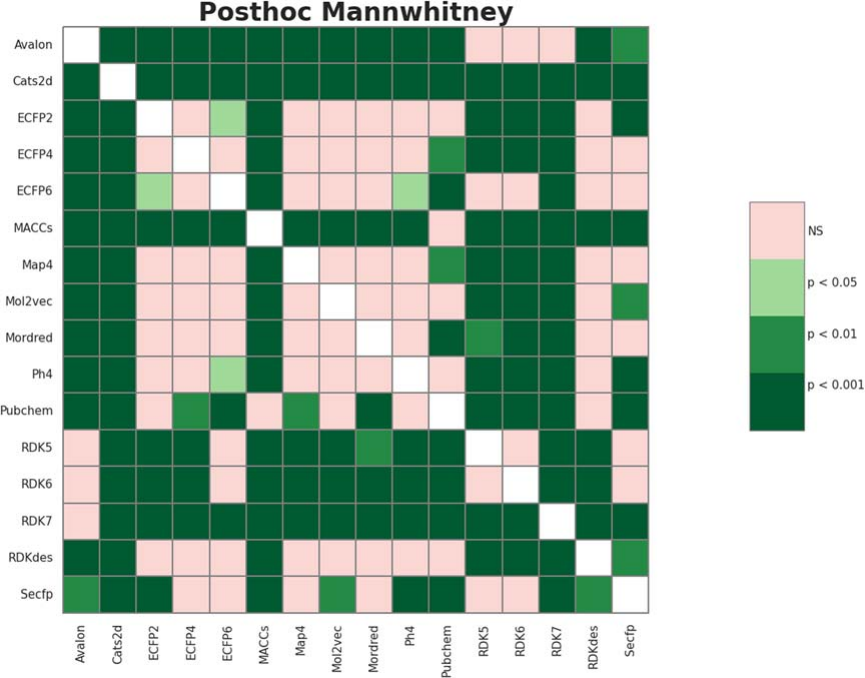
The heatmap illustrated the Wilcoxon signed-rank test 16 types of fingerprints and descriptors for meta-analysis.

From Fig. 2, the RDK7 fingerprint experienced the highest average F1-score in accordance with cross-validation (0.811), calculated from 10 models with 300 observations the whole. Meta-analysis was also conducted for pairwise comparison among fingerprints and descriptors using the Wilcoxon signed-rank test, illustrated in Table S2 (ESI).† As shown in Fig. 3, RDK7 showed a statistically higher significance of average F1-score compared to other datasets (*p* < 0.05). Therefore, RDK7 was selected as the optimal molecular feature to develop a machine learning model.

### 3.2. Model selection

#### 3.2.1 Feature extraction

**Fig. 4:**
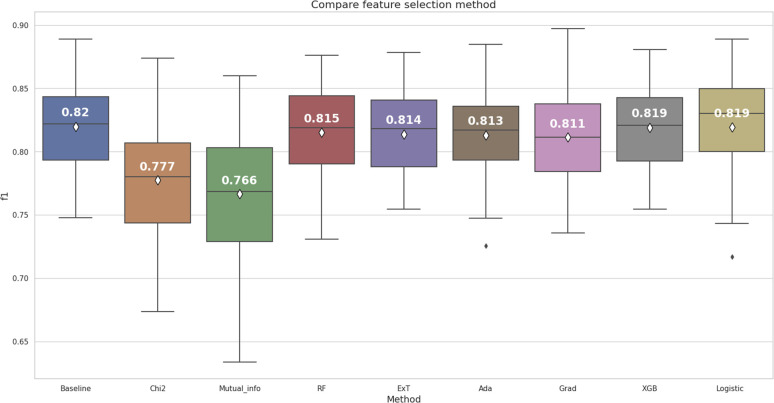
The feature extraction methods comparision for RDK7 dataset.

In this stage, our main objective was to select the optimal subset of RDK7 fingerprint, building the prediction model based on two criteria. Firstly, the model used for selection had to achieve the highest average F1-score in cross-validation with significant differences based on the Wilcoxon signed-rank test. Secondly, the model used for feature selection should yield the result with the minimum number of fingerprints to accelerate the optimization step. Fig. 4 illustrates the results of internal cross-validation among the fingerprint selection methods.

According to the box and whisker plot in Fig. 4, feature selection methods were stable except for mutual information, Logistic Regression, and AdaBoost returning F1-score outliers after 30 times cross-validation. While using the Wilcoxon signed-rank test for F1-score comparison among 8 models, only the Chi-squared and Mutual information gave statistically significantly lower results than the baseline model (*p* < 0.05). Other models had no statistically significant difference compared to the baseline model, so the feature extraction methods did not meet the first criterion.

On the other hand, the XGBoost and the Logistic Regression achieved the highest average F1-score among all the models, except for the baseline model. However, according to pairwise assessment of these two models (Fig. S1 ESI†), there was no statistically significant difference (*p* > 0.05). The second criterion, aimed at reducing computational resources by minimizing the number of features, was taken into consideration. The XGBoost algorithm had 533 bits, a lower number of features than the Logistic Regression algorithm, which had 744 bits. Therefore, the XGBoost algorithm was selected to reduce the dimension of the RDK7 dataset. The results of the features selection comparison are illustrated in Table S3 (ESI†).

#### 3.2.2 Machine learning model selection

Ten different machine learning algorithms were employed, and internal cross-validation along with the Wilcoxon signed-rank test was utilized to identify the most efficient machine learning model based on two criteria. The first criterion focused on selecting the model with an average F1-score derived from cross-validation that was significantly higher than the other models. As for the second criterion, the model with an average precision (AP) derived from cross-validation showed significantly higher performance compared to the other models and required a shorter training time.

**Fig. 5:**
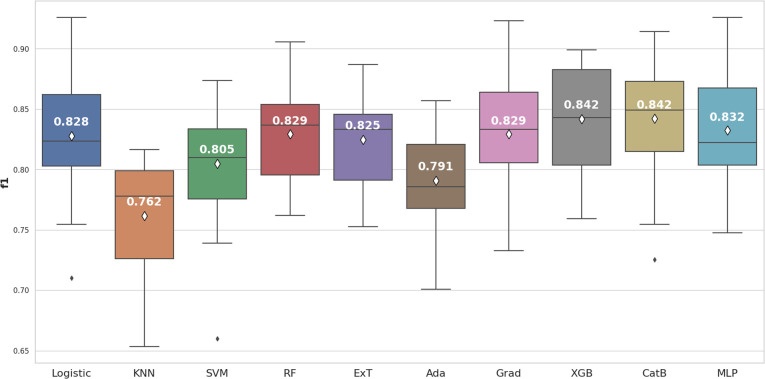
The machine learning algorithms comparision for RDK7 dataset utilizing F1-score.

**Fig. 6:**
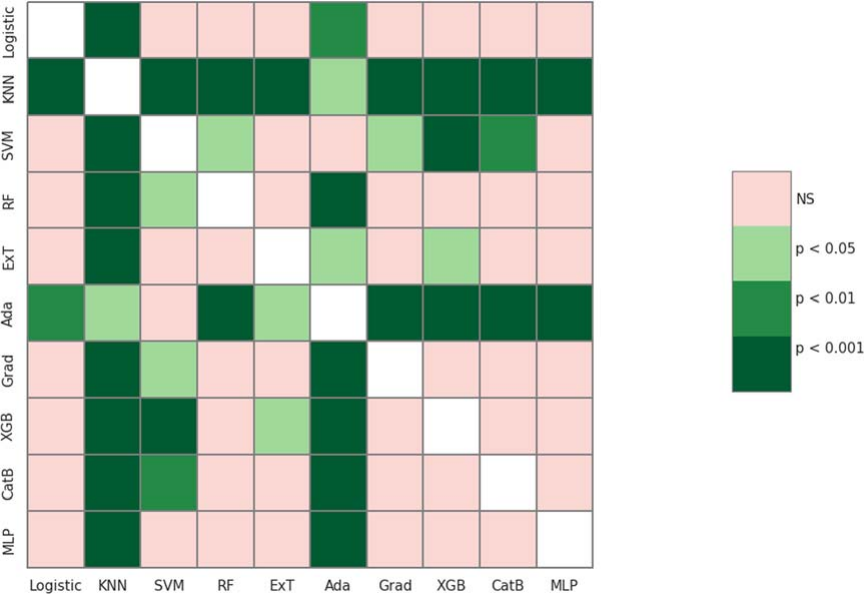
The Wilcoxon signed-rank test compared 10 machine learning algorithms using F1-score.

Based on the box and whisker plot in Fig. 5, XGBoost (0.842 ± 0.039) and CatBoost (0.842 ± 0.044) achieved the highest average F1-score. However, when the Wilcoxon signed-rank test was applied, these differences were not statistically significant (*p* > 0.05) compared to Logistic Regression, Random Forest, Gradient Boosting, and Multilayer Perceptron (Fig. 6).

**Fig. 7:**
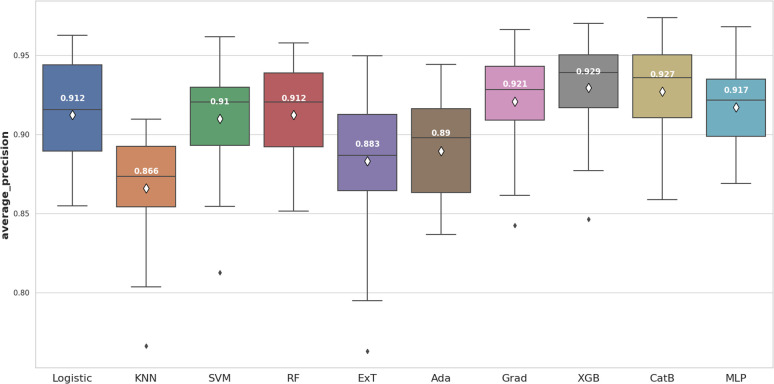
The machine learning algorithms comparision for RDK7 dataset utilizing average precision score.

We continued to use average precision (AP) to evaluate model performance (Fig. 7). Two models, including XGBoost (0.929 ± 0.029) and CatBoost (0.927 ± 0.030) remained the best performers. In this case, XGBoost showed significant differences when compared to most of the others (*p* < 0.05). In addition, XGBoost had a shorter training time than CatBoost. Thus, in terms of the second criterion for this step, XGBoost model was selected for the optimization.

#### 3.2.3 Machine learning model optimization

In the study, hyperparameters were optimized using Bayesian Optimization through 1000 trials. After analyzing the results, it was found that the highest average F1-score across all trials in the cross-validation was 0.854. Therefore, the hyperparameters associated with this trial were selected to be used for the XGBoost model. The results of this step are shown in Table S4 (ESI†).

The results derived from the cross-validation and external validation were consistent. This external validation result was highly generalizable and can be applied in virtual screening.

#### 3.2.4 Evaluating the generalizability of the model

The external validation dataset (20%) divided from the beginning was used to evaluate the generalizability of the model. The results are illustrated in Table 1.

**Table 1:** Internal and external validation results for the Gradient Boosting model

	Cross-validation			External validation		
	AP	F1-score	Recall	AP	F1-score	Recall
Baseline	0.929 ± 0.030	0.842 ± 0.038	0.835 ± 0.045	0.921	0.856	0.878
Optimize	0.928 ± 0.027	0.848 ± 0.041	0.855 ± 0.052	0.921	0.889	0.921

**Fig. 8:**
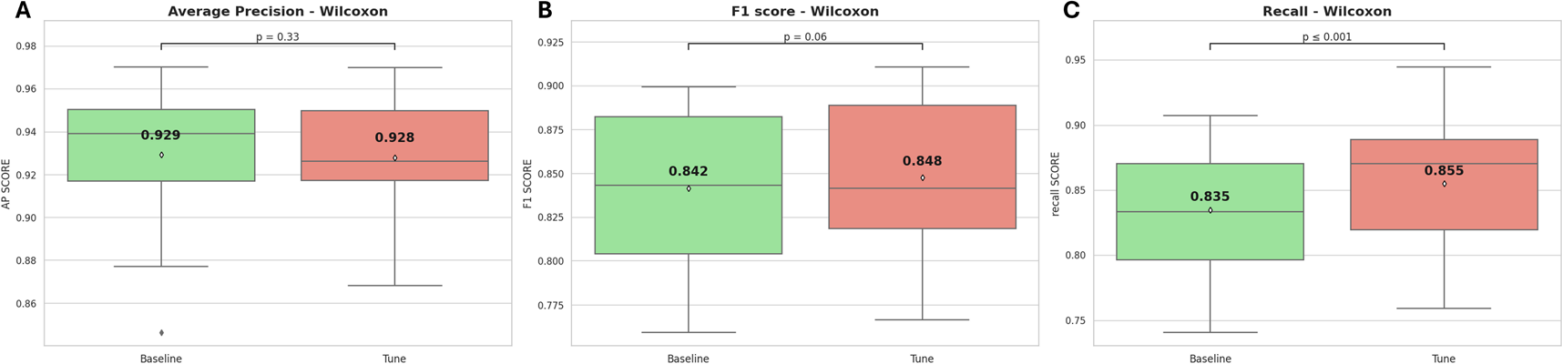
The internal cross-validation results of the model, both before and after hyperparameter optimization, (A) average precision, (B) F1-score, (C) recall.

According to Fig. 8, the CV-recall values are significantly higher after optimization than the default hyperparameter model, with a *p*-value ≤ 0.01. However, the average CV-AP and CV-F1 scores do not show a statistically significant improvement after optimization, with *p*-values of 0.33 and 0.06 respectively.

**Fig. 9:**
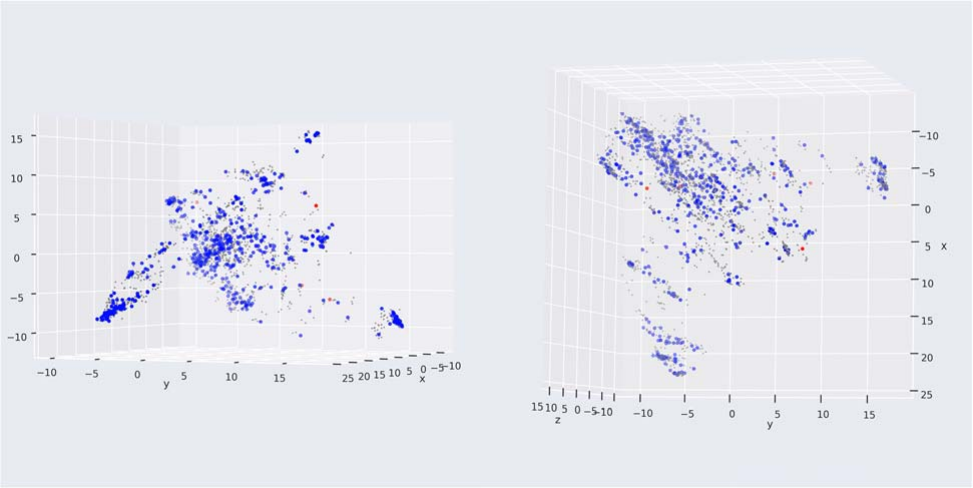
Application of LOF in applicability domain. The grey, blue, and red points describe the training set, external validation set in and out the applicability domain.

Compared with the study of L. A. Machado *et al.*,^16^ a state-of-the-art machine learning model targeting HIV integrase utilizing Mordred descriptor, our model could not outperform in external validation, with F1-score being 0.89 lower than the 0.93 of their study. However, our model development procedure is more rigid, with several decision-making stages, supported by the Wilcoxon signed-rank test of cross-validation. Moreover, our study performed cross-validation in the development pipeline for selection and optimization. At the same time, external validation was conducted in the final stage to prove the generalization of the machine learning model. The applicability domain was also investigated to remove five substances in the external validation set to ensure the interpolation of our model (Fig. 9). From Fig. 9 the red point was detected as a novelty, or outside applicability domain, which was far from the training set (grey points). This could solve the problem of sparse space in the bounding box approach.

### 3.3. Molecular docking

**Fig. 10:**
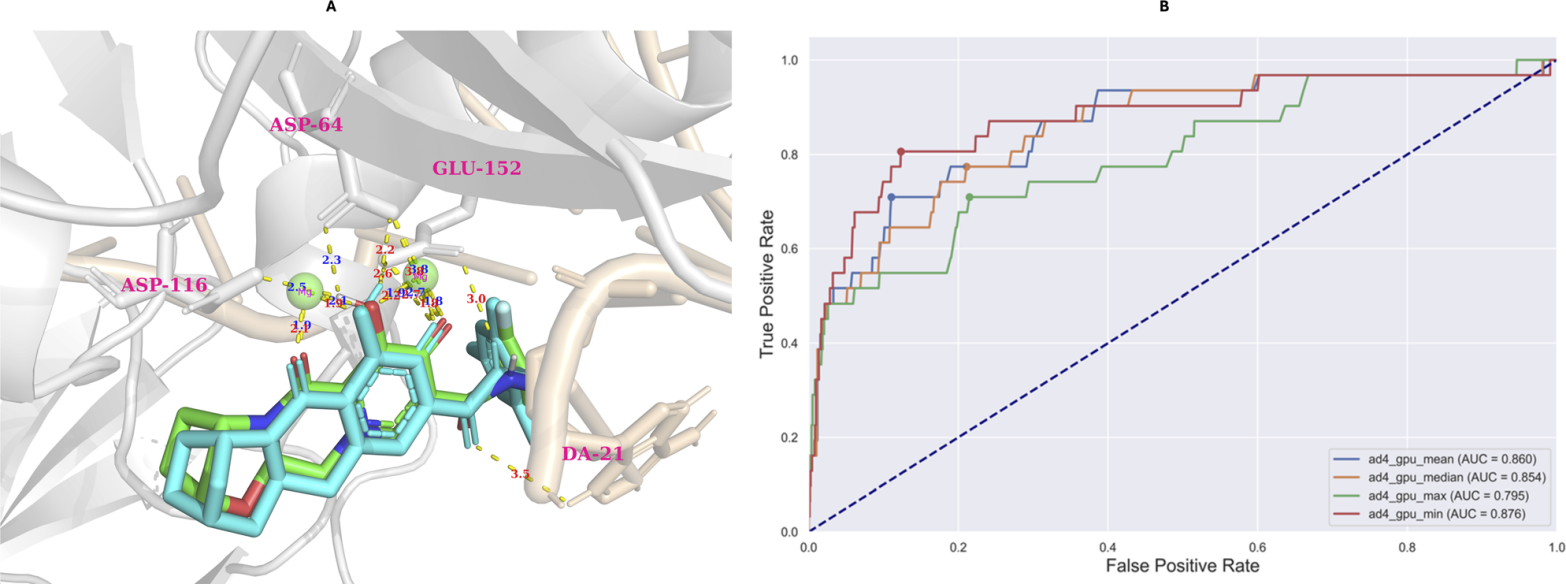
The results of redocking (A) and retrospective control (B) evaluation. The retrospective control was conducted utilizing four types of conformations, including the most negative (ad4_gpu_min), the most positive (ad4_gpu_max), the median (ad4_gpu_median), and mean (ad4_gpu_mean) of docked conformation distribution.

The model evaluation was conducted based on redocking data collected from Autodock-GPU.^35^ Based on the largest cluster of the redocking procedure, which comprised approximately 20% of all generated conformations, the RMSD value of the best-docked conformation (the most negative) did not exceed 2 Å (0.63 Å), which is illustrated in Fig. 10A.

According to the AUC-ROC curve in Fig. 10B, the AUC value was 0.876 for the most negative conformation (ad_gpu_min) with the G-Mean value reaching 0.841. The docking threshold extrapolated from the G-Mean was −9.71 kcal mol^−1^.

### 3.4. Virtual screening process

**Fig. 11:**
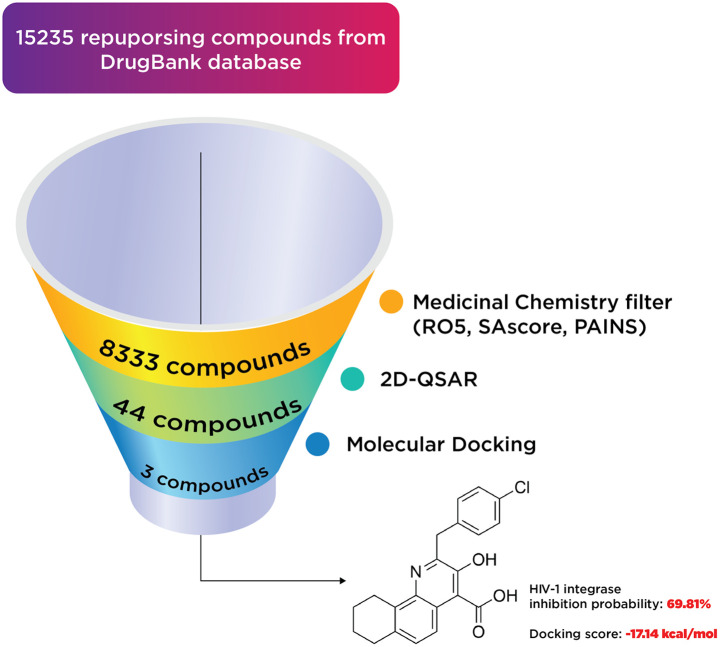
The results of virtual screening process.

A total of 15 235 substances from DrugBank were subjected to a medicinal chemistry filter, incorporating Lipinski's Rule^36^ of 5 (RO5 = 4), SAscore,^37^ and PAINS,^38^ yielding 8333 structures. Subsequent screening through a 2D-QSAR classification model identified 44 compounds as active. These compounds were further analyzed using molecular docking, leading to the identification of three notable compounds: two existing medicines and one hit compound (PSI-697). The outcomes of this virtual screening process are depicted in Fig. 11.

**Fig. 12:**
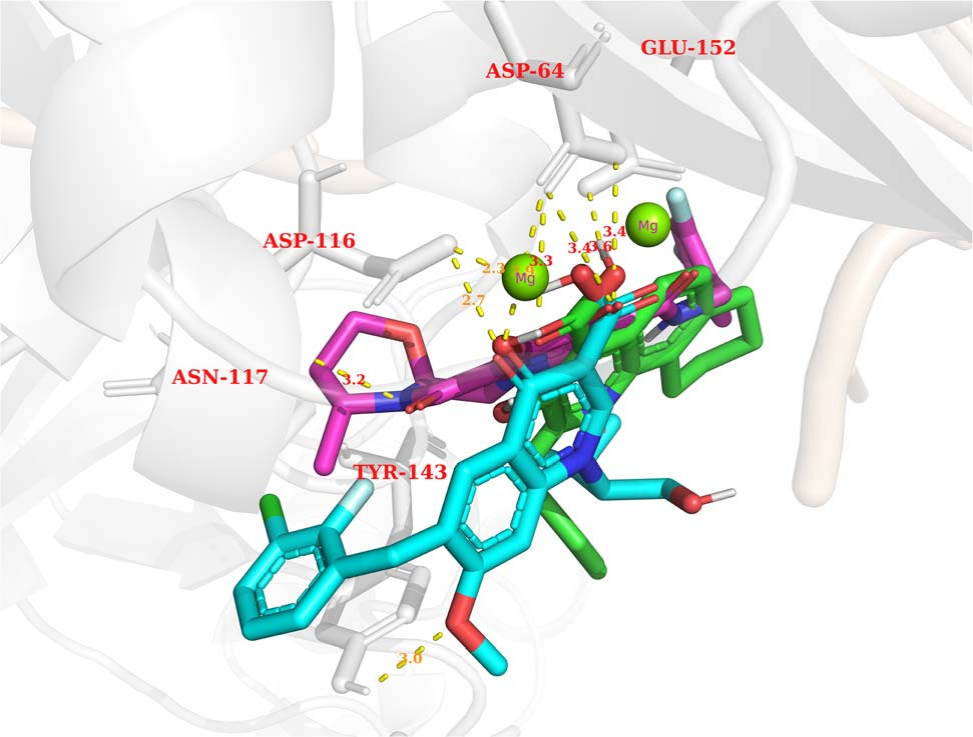
The binding modes of three potential candidates from the QSAR model. Blue: Elvitegravir (−17.32, 98,82%). Red: Dolutegravir (−11.42, 98,63%). Green: PSI-697 (−17.14, 69,81%).

In general, the results obtained from the molecular docking process showed that all three compounds in Fig. 12 formed electrostatic interactions with both Mg^2+^ ions. This is the first and highly important pharmacophore characteristic shared by all currently available INSTIs on the market. Additionally, all three selected conformations formed hydrogen bonds with the sidechain carbonyl group of Asp64, while fitting within the binding pocket between the two subunits of HIV integrase (matching the binding site of Bictegravir in the initial protein-ligand complex with PDB ID 6PUW).

**Fig. 13:**
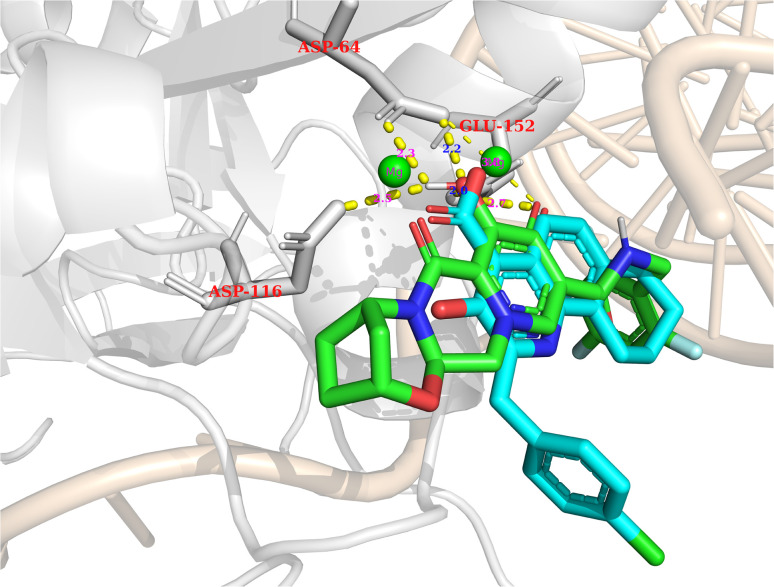
Bictegravir (red) and PSI-697 (green) in the active site.

Regarding PSI-697 (green) in Fig. 13, the binding mode was similar to Bictegravir, but the docking score was more negative, with the figures being −17,14 kcal mol^−1^ and −11 kcal mol^−1^, respectively. This could be explained by the hydrogen bonds with the sidechain of Glu152, which was observed in the complex of Bictegravir and protein. Moreover, the docking score of PSI-697 was also approximately equal to Elvitegravir (−17,32 kcal mol^−1^). The HIV integrase inhibitory probability of PSI-697 from the QSAR model was also good, with the figure being around 69%. As a result, PSI-697 was the most promising candidate targeting HIV integrase for both inhibition and binding ability.

## 4. Conclusion

Our study introduced a novel approach to machine learning, where decision-making stages were made based on statistical tests. We utilized 16 different molecular fingerprints and descriptors and employed the Wilcoxon signed-rank test of cross-validation to determine the optimal one for feature and model selection. The LOF algorithm was implemented to establish the applicability domain, outperforming the bounding box technique in sparse areas.

The RDK7 fingerprint proved the most suitable and XGBoost was the best model. External validation yielded impressive results with an F1-score of 0.889, average precision of 0.921, and recall of 0.921. These findings are highly generalizable and valuable for the virtual screening of potential HIV-1 integrase inhibitors.

The repurposing structure library was screened, resulting in the identification of one potential compound. We recommend the synthesis and biological activity testing of this potential compound.

## Data availability

The source code, notebooks, and all datasets are available at: https://github.com/Medicine-Artificial-Intelligence/HIV_IN_Classification_ML.

## Author contribution

T. L. P played a key role in the experiment by working on the source code and significantly contributing to writing and organizing the manuscript. T. C. T and V. T. T were tasked with developing a QSAR (Quantitative Structure–Activity Relationship) model. T. A. P and P. C. V. N took on the responsibilities of collecting data, analyzing it to find meaningful patterns, and handling the graphical representation of molecular docking. T. M. P managed to compile the package and uploaded it to Github for public access. Additionally, V. T. T contributed to the analysis and helped develop the source code for molecular docking. T. N. T provided insightful suggestions, helped improve the manuscript, and played a guiding role in the project's direction. All the team members thoroughly reviewed and gave their approval for the final version of the research paper.

## Conflicts of interest

There are no conflicts of interest to declare.

## Supplementary Material

RA-014-D4RA02231A-s001

## References

[cit1] Van Heerden A., Humphries H., Geng E. (2023). Curr. Opin..

[cit2] Furman P. A., Fyfe J. A., St Clair M. H., Weinhold K., Rideout J. L., Freeman G. A., Lehrman S. N., Bolognesi D. P., Broder S., Mitsuya H. (1986). Proc. Natl. Acad. Sci. U. S. A..

[cit3] De Clercq E. (1998). Antivir. Res..

[cit4] Mahdi M., Mótyán J. A., Szojka Z. I., Golda M., Miczi M., Tőzsér J. (2020). Virol. J..

[cit5] McColl D. J., Chen X. (2010). Antivir. Res..

[cit6] Ray N., Blackburn L. A., Doms R. W. (2009). J. Virol..

[cit7] Blanco J. L., Whitlock G., Milinkovic A., Moyle G. (2015). Expert Opin. Pharmacother..

[cit8] Stern T. A. , Fricchione G. L. and Rosenbaum J. F., Massachusetts General Hospital Handbook of General Hospital Psychiatry-E-Book, Elsevier Health Sciences, 2010

[cit9] Pau A. K., George J. M. (2014). Clin. Infect. Dis..

[cit10] Baptista D., Ferreira P. G., Rocha M. (2021). Briefings Bioinf..

[cit11] Zhang L., Zhang H., Ai H., Hu H., Li S., Zhao J., Liu H. (2018). Curr. Top. Med. Chem..

[cit12] Kong J., Lee H., Kim D., Han S. K., Ha D., Shin K., Kim S. (2020). Nat. Commun..

[cit13] Ståhl N., Falkman G., Karlsson A., Mathiason G., Bostrom J., Chemi J. (2019). Inf. Model..

[cit14] Li Y., Wu Y., Yan A. (2017). Mol. Inform..

[cit15] Kurczyk A., Warszycki D., Musiol R., Kafel R., Bojarski A. J., Polanski J., Chemi J. (2015). Inf. Model..

[cit16] Machado L. A., Krempser E., Guimarães A. C. R. (2022). Front. Drug Discov..

[cit17] Gad S. C. , in Encyclopedia of Toxicology, ed. P. Wexler, Academic Press, Oxford, 3rd edn, 2014, pp. 1–9

[cit18] McCrum-Gardner E. (2008). Br. J. Oral Maxillofac. Surg..

[cit19] Landrum G. (2013). Release.

[cit20] Moriwaki H., Tian Y.-S., Kawashita N., Takagi T. (2018). J. Cheminf..

[cit21] Alice Capecchi T. , Richard Gowers, 2022, Map4, https://github.com/reymond-group/map4, (accessed Nov 13)

[cit22] Probst D., Reymond J.-L. (2018). J. Cheminf..

[cit23] Kramer O. , in Machine Learning for Evolution Strategies, Springer, 2016, pp. 45–53

[cit24] Holm S. (1979). Scand. J. Stat..

[cit25] Terpilowski M. A. (2019). J. Open Source Softw..

[cit26] Demšar J. (2006). J. Mach. Learn. Res..

[cit27] Kuss M., Rasmussen C. E., Herbrich R. (2005). J. Mach. Learn. Res..

[cit28] Nguyen V. , IEEE Second International Conference on Artificial Intelligence and Knowledge Engineering (AIKE), 2019

[cit29] Ho S. Y., Phua K., Wong L., Goh W. W. B. (2020). Patterns.

[cit30] Fernández A. , García S., Galar M., Prati R. C., Krawczyk B. and Herrera F., Learning from Imbalanced Data Sets, Springer, 2018

[cit31] Davis J. and Goadrich M., Proceedings of the 23rd International Conference on Machine Learning, 2006, 10.1145/1143844.1143874

[cit32] Passos D. O., Li M., Jóźwik I. K., Zhao X. Z., Santos-Martins D., Yang R., Smith S. J., Jeon Y., Forli S., Hughes S. H. (2020). J. Sci..

[cit33] Imrie F., Bradley A. R., Deane C. M. (2021). Bioinformatics.

[cit34] Akosa J. , Proceedings of the SAS Global Forum, 2017, vol. 12, pp. 1–4

[cit35] Santos-Martins D., Solis-Vasquez L., Tillack A. F., Sanner M. F., Koch A., Forli S. (2021). J. Chem. Theory Comput..

[cit36] Lipinski C. A., Lombardo F., Dominy B. W., Feeney P. J. (2012). Adv. Drug Deliv. Rev..

[cit37] Ertl P., Schuffenhauer A. (2009). J. Cheminf..

[cit38] Baell J. B., Holloway G. A. (2010). J. Med. Chem..

