# Supplementary material for: Novel machine learning approach toward classification model of HIV-1 integrase inhibitors
Source: RSC Adv. 2024 May 2;14(21):14506–13. doi: 10.1039/d4ra02231a (PMC11064125; doi:10.1039/d4ra02231a)
Supplement: RA-014-D4RA02231A-s001 [file RA-014-D4RA02231A-s001.pdf]

Electronic Supplementary Material (ESI) for RSC Advances.
This journal is © The Royal Society of Chemistry 2024

Novel machine learning approach toward classification model of HIV-1 integrase inhibitors

Tieu-Long Phan[a, b], The-Chuong Trinh[c], Van-Thinh To[d], Thanh-An Pham[d], Phuoc-Chung Van Nguyen[d], Tuyet-Minh Phan[d], Tuyen Ngoc Truong[*d]

Corresponding Author

***Tuyen Ngoc Truong,** Email: truongtuyen@ump.edu.vn. Phone: +84 903330604.

[a]Department of Organic Chemistry, Faculty of Pharmacy, University of Medicine and Pharmacy at Ho Chi Minh City, Ho Chi Minh City, Vietnam.

Data Availability: All datasets used in our research could be downloaded freely from the ChEMBL database, the raw data and virtual screening data are available online at https://github.com/Medicine-Artificial-Intelligence/HIV_IN_Classification_ML/tree/main/Data. 16 different molecular descriptor and fingerprint were calculated by this notebook.

Table S1. Compare processing and feature selection of 16 types of data

Table S1. Compare processing and feature selection of 16 types of data

	Number of features	Number of features after threshold analysis of variance	Number of outliers	Number of features selected
**Avalon**	1025	801	118	221
**Cats2d**	211	174	195	53
**ECFP2**	2049	99	0	28
**ECFP4**	2049	151	0	51
**ECFP6**	4097	170	6	56
**MACCs**	168	109	3	38
**Map4**	1025	1022	0	230
**Mol2vec**	300	301	43	89
**Mordred**	1613	793	36	221

**Ph4**	39973	1593	86	454
**Pubchem**	882	291	32	82
**RDK5**	2049	1443	56	361
**RDK6**	2049	2003	78	518
**RDK7**	4097	4059	112	954
**RDKdes**	209	140	78	53
**Secfp**	2049	245	1	74

Table S2. Wilcoxon signed rank test for meta-analysis of 16 datasets

Table S2. Wilcoxon signed rank test for meta-analysis of 16 datasets

	Avalon	Cats2d	ECFP2	ECFP4	ECFP6	MACCs	Map4	Mol2vec	Mordred	Ph4	Pubchem	RDK5	RDK6	RDK7	RDKdes	Secfp
**Avalon**	1.000	0.000	0.000	0.000	0.000	0.000	0.000	0.000	0.000	0.000	0.000	0.813	0.870	0.009	0.000	0.021
**Cats2d**	0.000	1.000	0.000	0.000	0.000	0.000	0.000	0.000	0.000	0.000	0.000	0.000	0.000	0.000	0.000	0.000
**ECFP2**	0.000	0.000	1.000	1.000	0.014	0.000	0.241	1.000	0.870	1.000	0.208	0.000	0.000	0.000	1.000	0.000
**ECFP4**	0.000	0.000	1.000	1.000	0.871	0.000	1.000	1.000	1.000	1.000	0.000	0.001	0.000	0.000	1.000	0.022
**ECFP6**	0.000	0.000	0.014	0.871	1.000	0.000	1.000	0.043	1.000	0.008	0.000	0.235	0.109	0.000	0.098	0.349
**MACCs**	0.000	0.000	0.000	0.000	0.000	1.000	0.000	0.000	0.000	0.000	1.000	0.000	0.000	0.000	0.000	0.000
**Map4**	0.000	0.000	0.241	1.000	1.000	0.000	1.000	1.000	1.000	0.720	0.000	0.003	0.000	0.000	1.000	0.463
**Mol2vec**	0.000	0.000	1.000	1.000	0.043	0.000	1.000	1.000	1.000	1.000	0.001	0.000	0.000	0.000	1.000	0.000
**Mordred**	0.000	0.000	0.870	1.000	1.000	0.000	1.000	1.000	1.000	0.219	0.000	0.007	0.000	0.000	1.000	0.159
**Ph4**	0.000	0.000	1.000	1.000	0.008	0.000	0.720	1.000	0.219	1.000	0.019	0.000	0.000	0.000	1.000	0.001
**Pubchem**	0.000	0.000	0.208	0.000	0.000	1.000	0.000	0.001	0.000	0.019	1.000	0.000	0.000	0.000	0.040	0.000
**RDK5**	0.813	0.000	0.000	0.001	0.235	0.000	0.003	0.000	0.007	0.000	0.000	1.000	1.000	0.000	0.000	1.000
**RDK6**	0.870	0.000	0.000	0.000	0.109	0.000	0.000	0.000	0.000	0.000	0.000	1.000	1.000	0.000	0.000	0.463
**RDK7**	0.009	0.000	0.000	0.000	0.000	0.000	0.000	0.000	0.000	0.000	0.000	0.000	0.000	1.000	0.000	0.000
**RDKdes**	0.000	0.000	1.000	1.000	0.098	0.000	1.000	1.000	1.000	1.000	0.040	0.000	0.000	0.000	1.000	0.000
**Secfp**	0.021	0.000	0.000	0.022	0.349	0.000	0.463	0.000	0.159	0.001	0.000	1.000	0.463	0.000	0.000	1.000

*$p = 0.000$ mean $p < 0.00$

Table S3. The results of features selection comparison

Table S3. The results of features selection comparison

Model	Number of fingerprints	Mean	Median	Outliers	Compared to baseline
**Baseline**	4078	0.820	0.822	0	
**Chi2**	20	0.777	0.780	0	p < 0,05
**Mutual**	20	0.766	0.766	1	p < 0,05
**RF**	954	0.815	0.819	0	NS
**ExT**	924	0.814	0.818	0	NS
**ADA**	50	0.813	0.817	4	NS
**Grad**	293	0.811	0.811	0	NS
**XGB**	533	0,819	0,821	0	NS
**Logic**	744	0,819	0,830	0	NS

*NS: not significant

Table S4. Hyperparameters of optimization step

Table S4. Hyperparameters of optimization step

Hyperparameters	Values
**sampling_strategy**	0.6080363095456893
**max_depth**	5
**min_child_weight**	1
**learning_rate**	0.13853526499953608
**n_estimators**	54
**gamma**	0.0016145215090081605
**reg_alpha**	3.3173836339552476e-07
**reg_lambda**	5.833565947119527e-06
**subsample**	0.8893106963312789
**colsample_bytree**	0.6351067897414812

Figure S1. Feature selection models comparison using the Wilcoxon signed-rank test

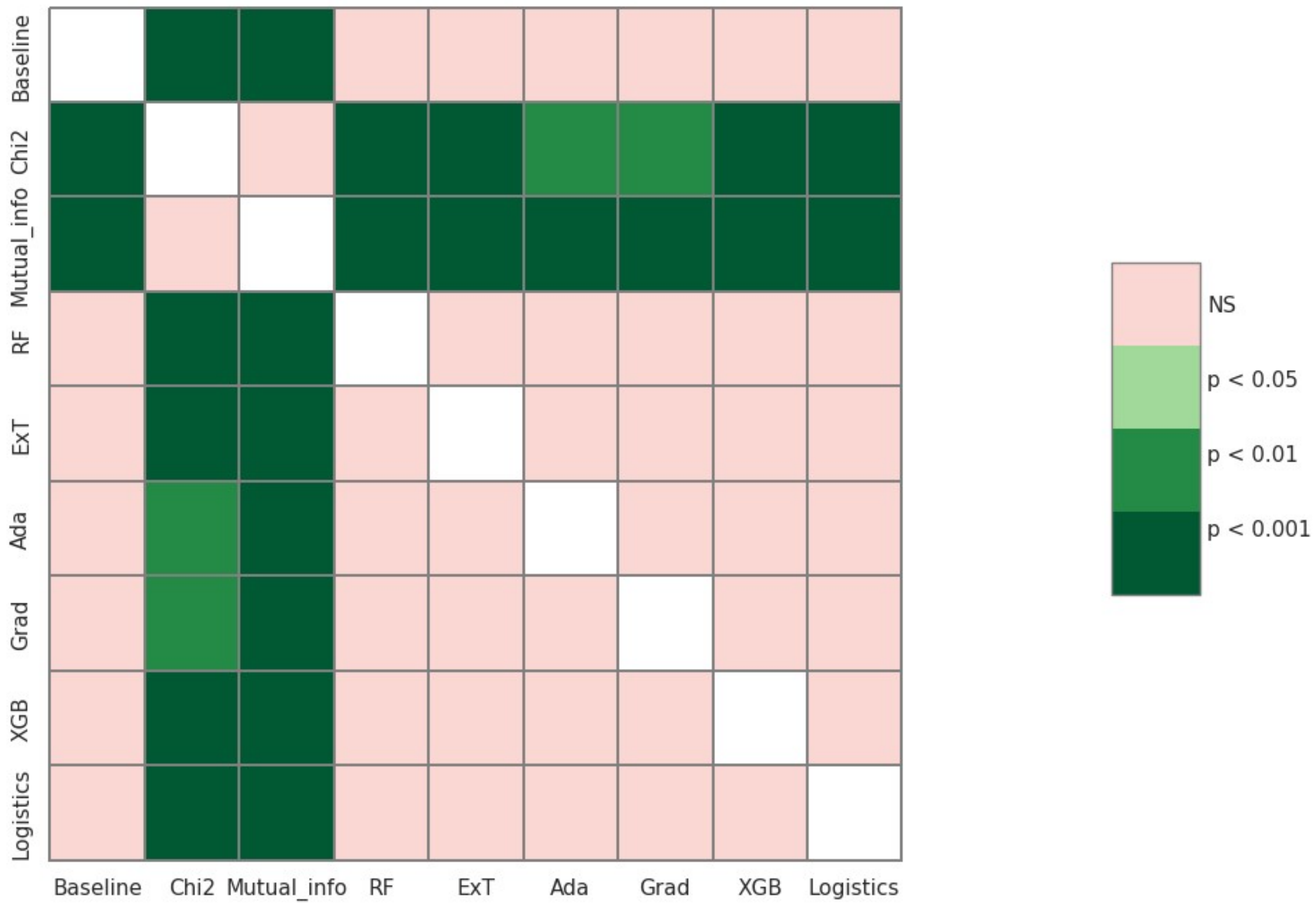

Figure S1. Feature selection models comparison using the Wilcoxon signed-rank test
